# Performance of Frontloading for Smear Microscopy in the Diagnosis of Pulmonary Tuberculosis: A Cross-Sectional Study at a Referral Hospital in Uganda

**DOI:** 10.1371/journal.pone.0048531

**Published:** 2012-10-29

**Authors:** Penelope Miremba, Joan N. Kalyango, William Worodria, Henry Mugerwa, Ethel Nakakawa, Benon B. Asiimwe

**Affiliations:** 1 Clinical Epidemiology Unit, College of Health Sciences, Makerere University, Kampala, Uganda; 2 Department of Pharmacy, College of Health Sciences, Makerere University, Kampala, Uganda; 3 Department of Medicine, Mulago Hospital Complex, Kampala, Uganda; 4 Department of Medical Microbiology, College of Health Sciences, Makerere University, Kampala, Uganda; University College Dublin, Ireland

## Abstract

**Objective:**

To compare the performance of frontloading and the standard WHO method for diagnosis of pulmonary TB at Mulago Hospital in order to validate the technique in this setting.

**Methods:**

This was a cross-sectional study in which 229 adult (≥18 years) TB suspects were consecutively enrolled. Suspects submitted three sputum samples as follows: at initial presentation, one hour after the first sample, and the next morning. The first and next morning samples formed the standard WHO method, while the first and the one hour later samples formed the frontloading method. Sample processing was by the standard N-acetyl L-cystein (NALC)-NaOH method, and fluorescent microscopy was done for both methods, while cultures of the first sample on Lowenstein-Jensen slants acted as a gold standard. The sensitivity, specificity and predictive values for the WHO standard and frontloading methods were compared.

**Results:**

The sensitivity of both the frontloading and standard schemes was 91.1% while their specificities were 86.2% and 91.7% respectively. There was excellent agreement between the diagnostic capacity of the two methods (*kappa* statistic = 0.87, P<0.0001). The positive predictive value for the frontloading scheme was 87.2% and that for the standard approach was 91.9%, while the negative predictive values were 90.4% and 90.9%, respectively. Among the HIV positive patients, frontloading identified 59/79 (74.7%) culture positive samples while the standard approach identified 55/79 (69.6%). In the HIV sero-negative category, on the other hand, front-loading identified 48/110 (43.6%) culture positive samples compared to 45/110 (40.9%) by the standard approach.

**Conclusion:**

Frontloading based on smear examination of two same-day sputum samples has a similar performance to the current standard method and would not be associated with any significant missed diagnosis. It may therefore be advocated for use in our setting so as to reduce time to completion of diagnosis and patient loss to follow-up.

## Introduction

Tuberculosis (TB) is one of the most common infectious causes of mortality in the world with control in a majority of the patients, who are mostly in resource-limited settings, hindered by many economic barriers that include costly pathways to diagnosis [1]. Diagnosis of pulmonary tuberculosis (PTB) in low and middle income countries greatly depends on smear microscopy, using 2 sputum samples. The WHO policy recommends the 2 sputum samples to be collected as spot-morning samples [2]. In this standard approach, the first sample is collected at the time of consultation and the second sample is an early morning sample provided on the next morning. However, the time from collection of the first sample to second is not specified by the WHO. Consequently, patients must return to the clinic over two days to provide the recommended two sputum samples and many do not return due to distance and financial constraints, hence do not receive treatment due to drop-out. Moreover, because of poor radiological services, many places in Uganda still do up to three sputum samples before starting treatment, further compounding this dilemma [3]. Another technique, referred to as front-loading, has been proposed where two sputum samples are collected and analyzed on the same day [4]. Front-loading would offer immediate results, help on treatment decision making and hence reduce on the dropout rate and ultimately control disease spread. This technique was be able to diagnose from 76% to 97% patients with PTB in two different studies, comparable to the standard scheme which identifies from 73% to 97% [5], [6], while a multi-country study found the sensitivity of the dame technique at 63.6% (95% CI 59.7%–67.5%), which was non-inferior to spot-morning (64.8%, 95% CI 61.3%–68.3%) recommended by the WHO [7].

Liquid and solid based media for culture for *Mycobacterium tuberculosis* complex require several days (liquid culture) to several weeks (solid culture) for growth. Newer technologies for diagnosis of TB like the Xpert MTB/RIF, which have a high performance and are recommended for wide use in HIV-associated and drug-resistant TB are expensive and not yet available for routine use in low resource settings. Therefore optimization of smear microscopy as a diagnostic tool is important for many high disease-burden low resource settings, since absence of prompt diagnosis will lead to continued transmission of infection in communities. The current study aimed at determining the performance (sensitivity and specifity) of the front-loading technique for same day microscopic diagnosis of suspect sputum samples so as to compare the results with those obtained with the standard WHO scheme, using sputum culture as the gold standard.

**Table 1:** Socio-demographic characteristics of 221 patients who attended Assessment Centre and the TB clinic, Mulago Hospital complex, between January and April inclusive, 2011.

Characteristic		Number	Proportion (%)
**Age (years)**	<20	05	2.3
	20–29	77	34.8
	30–39	65	29.4
	40–49	32	14.5
	50+	19	8.6
	NA	23	10.4
**Marital status**	Never married	109	49.3
	Married	92	41.6
	Divorced	20	9.1
**Sex**	Male	135	61.1
	Female	86	38.9
**HIV**	Positive	79	35.7
	Negative	110	49.8
	Not done	32	14.5
**Cough**	2 weeks –2 months	101	45.7
	>2 months	120	54.3
**Chest pain**	Yes	165	74.7
	No	56	25.3
**Night sweats**	Yes	133	60.2
	No	88	39.8
**Anorexia**	Yes	105	47.5
	No	116	52.5
**Weight loss**	Yes	120	54.3
	No	101	45.7
Treatment history *	Yes	18	08.1
	No	203	91.9

NA = information not available *Prior history of TB treatment, not currently on treatment.

## Materials and Methods

### Ethics Statement

Ethical approval was obtained from Makerere University School of Medicine Research and Ethics Committee and the Uganda National Council of Science and Technology. All study participants provided written informed consent. All patients with culture positive results and not yet on TB therapy were recalled for treatment.

**Table 2:** Performance of frontloading and WHO standard scheme for 221 patients, at the National referral hospital, Mulago.

Technique	Smearresult	Culturepositive	Culturenegative	Total
**Front-loaded**	Positive	102*	15*	117
	Negative	10	94	104
	Total	112	109	221
**Standard scheme**	Positive	102*	09*	111
	Negative	10	100	110
	Total	112	109	221

* At least one smear was positive.

**Table 3:** Performance of the three samples by HIV status.

Category	Smear status	HIV status			
		Positive (%)	Negative (%)	Not done (%)	Total (%)
**Spot smear**	Positive	53 (51)	42 (40.3)	9 (8.7)	104 (100)
	Negative	26 (22.2)	68 (58.1)	23 (19.7)	117 (100)
**Xspot**	Positive	54 (52.9)	40 (39.2)	8 (7.9)	102 (100)
	Negative	25 (21)	70 (58.8)	24 (20.2)	119 (100)
**Morning**	Positive	49 (49.5)	39 (39.4)	11 (11.1)	99 (100)
	Negative	23 (22.6)	59 (57.8)	20 (19.6)	102 (100)
	Not done	7 (35)	12 (60)	1 (5)	20 (100)

The 1^st^ smear alone identified 117/221 as smear positive, the 2^nd^ (Xspot) smear identified 2 more making it 119/221 as smear positive, and the 3^rd^ smear identified 3 more in addition, making it 122/221 as positive.

### Study Design and Setting

A cross sectional study was conducted between January and April 2011 at Mulago National Referral and Teaching Hospital, Kampala, Uganda. The study was based at the assessment centre (a general outpatients unit of the hospital) and the tuberculosis clinic (which mainly recruits TB/HIV co-infected individuals). Sample processing, microscopy, and culture were performed at the biosafety level (BSL) 3 mycobacteriology laboratory, Department of Medical Microbiology, Makerere University College of Health Sciences.

### Study Participants and Sampling

At Ward 5 and 6 (TB/HIV clinic), patients were enrolled into the study if they were aged 18 years and above, presented with a cough of more than 2 weeks, and gave written informed consent to participate in the study. Patients on TB treatment or those who failed to expectorate sputum were excluded. A total of 231 suspects with a history of cough of more than two weeks were enrolled into the study using consecutive sampling. Two of the suspects were excluded from the study because they could not expectorate. At the clinic, two samples (spot and morning) were taken 229 suspects and direct smear microscopy performed. The smears were stained using Ziehl Neelsen (ZN) and/or Auramine-O staining method, depending on which reagents were available, and read by two trained laboratory technicians for quality assurance. Results from microscopy were used for patient management.

### Sample and Data Collection

We used a pre-tested questionnaire to obtain data on participants’ demographic characteristics including age, area of residence, sex and marital status; and clinical characteristics including symptoms and history of previous TB treatment. The study physician administered the questionnaire and requested patients to submit sputum specimens as follows: an immediate sample provided after the consultation, an additional sample collected one hour after the first one on the same day of presentation to the hospital as previously described [6], and an early morning sample the next day. Sputum specimens collected for the purpose of this study were refrigerated and transported within 48 hours to the BSL 3 mycobacteriology laboratory for processing and culture. The sample collected at initial presentation and the sample collected one hour after the first one comprised the frontloading scheme also referred to as the spot-spot scheme. The sample collected at initial presentation and the one collected the next morning comprised the WHO standard scheme also referred to as the spot-morning scheme.

### Sputum Sample Processing, Culture and Identification

Specimens (2.5–10 ml) were processed by the standard N-acetyl L-cystein (NALC)-NaOH method [8] and concentrated at 4000 × *g* for 15 minutes. The sediment, irrespective of the original sample volume, was reconstituted to 2.5 ml with phosphate buffer pH 6.8, to make the inoculums for the smears and cultures. The smears were stained using Auramine-O staining method, read by trained laboratory technicians and graded using the WHO/International Union against Tuberculosis and Lung Disease (IUATLD) scale. The grading of smears at the BSL3 laboratory was conducted while unaware of results of same patient from the recruitment clinic. All smears with at least 1 acid fast bacillus per 100 high power fields were considered positive. Two Lowenstein-Jensen slants were inoculated with part of the sediment from the first sample and incubated at 37°C. Cultures were considered negative when no colonies were seen after eight weeks of incubation as per standard guidelines [8]. Any number of colonies on culture was considered positive. Fluorescent smear microscopy on sediments was performed using Auramine-O staining. In addition, HIV serology was done for patients whose status was not known using STAT-PAK (Chembio, Medford, NY), and Determine HIV-1/2 (Abbott, Tokyo, Japan). The BSL3 mycobacteriology laboratory successfully participates in annual external proficiency testing programs organized by the Supra national laboratory net work.

### Data Analysis

The characteristics of the study participants were summarized with descriptive statistics including means, medians, standard deviations and ranges for numerical characteristics or percentages and frequencies for categorical variables. The sensitivities, specificities, negative predictive values (NPV) and positive predictive values (PPV) of sputum smear microscopy for both schemes were determined using culture as the gold standard. Their 95% confidence intervals were also determined, and the kappa statistic (with its *p*-value) was used to determine the level of agreement between the two schemes. In addition, the performance of the two schemes was compared among patients with and without HIV. *P*-values less than 0.05 were considered statistically significant. All analyses were done using STATA version 10 (College Station, Texas, USA).

## Results

### Socio-demographic and Clinical Characteristics of the Participants

We enrolled 229 patients into the study but only data from 221 participants was analyzed because eight of the samples did not give culture results. The median age of the participants was 30 years (Interquartile range [IQR] 25, 40). The participants were mostly males 135/221 (61.1%) and 79/221 (50.7%) were HIV positive. The main clinical symptoms that the patients presented with were chest pain (165/221, 74.7%), night sweats (133/221, 60.2%) and weight loss (120/221, 54.3%). Most patients (120/221, 54.3%) had a cough of a period greater than 2 months. About 8.1% of the patients had past history of TB treatment and were all sputum smear and culture negative. The socio-demographic characteristics of the participants are summarized in Table 1.

**Table 4a:** Sensitivity and specificity of frontloading versus standard sputum smears for diagnosis of TB in 221 patients, Mulago hospital, 2011; **Table 4b.** Negative and positive predictive values of frontloading versus standard sputum smears for diagnosis of TB in 204 patients, Mulago Hospital, 2011.

Table 4a				
Approach	Sensitivity	95% CI	Specificity	95% CI
**Frontloaded**	91.1%	84.2%, 95.6%	86.2%	78.3%, 92.1%
**Standard sputum smears**	91.1%	84.2%, 95.6%	91.7%	84.9%, 96.2%
PPV = Positive Predictive Value, NPV = Negative Predictive Value, CI = confidence interval				
**Table 4b**				
**Approach**	**PPV**	**95% CI**	**NPV**	**95% CI**
**Frontloaded**	87.2%	79.7%, 92.6%	90.4%	83.0%, 98.3%
**Standard sputum smears**	91.9%	85.2%, 96.2%	90.9%	83.9%, 95.6%

PPV = Positive Predictive Value, NPV = Negative Predictive Value, CI = confidence interval.

### Performance of Frontloading and the Standard Scheme

We included 221 patients in the evaluation of performance of the two schemes because eight (3.5%) of the participants enrolled did not have culture results due to heavy contamination and were thus excluded from the analysis. In addition, 20 (9%) patients did not return the next day to provide the second sample for the WHO standard scheme. Of these, six were smear positive on the first smear while four were culture positive. These subjects were however included in the analysis and the conclusion about their results was based on the first sample.

Smear microscopy using the frontloading scheme identified 117 (53%) patients with at least one positive smear. The smear from the first sample identified 104 (88%) positive subjects while the sample collected one hour after the first (that is the X spot) identified an extra 13 (11%) positive subjects. In comparison, smear microscopy using the WHO standard scheme identified 111 (50.2%) patients with at least one positive smear. The sample collected the next morning (i.e. the morning sample) identified an additional 7 (6%) patients with a positive smear. Both the front-loading and standard scheme identified 112 positive cultures (Table 2). Detailed information on the performance of the three samples by HIV status is shown in Table 3.

The sensitivity of both frontloading and the WHO standard scheme was 91%. The specificity of the frontloading scheme was 86.2% while that of the WHO standard scheme was 91.7% (Table 4a). The positive (PPV) and negative (NPV) predictive values by the frontloading technique was 87.2% and 90.4% respectively. The PPV for the standard approach was 91.9% while the NPV was 90.9% (Table 4b). The level of agreement (Kappa Statistic) between the two approaches was 0.87 (p<0.0001). Among the HIV positive patients, frontloading identified 59/79 (74.7%) culture positive samples while the standard approach identified 55/79 (69.6%). In the HIV sero-negative category, on the other hand, front-loading identified 48/110 (43.6%) culture positive samples compared to 45/110 (40.9%) by the standard approach.

## Discussion

Newer diagnostic technologies have been developed for the rapid diagnosis of TB, many of which are challenging to use outside of TB reference and research laboratories. TB control in resource poor, high disease-burden settings therefore continues to rely on smear microscopy which is associated with considerable patient costs and inconveniences because of the need to submit multiple sputum specimens over a period of up to three days [9], and a number of TB control programs have reported initial patient default as a result [10].

The sensitivity of front-loading technique in this study was identical to that of the standard approach, both at 91% (CI: 84.2%–95.6%). A previous study on a collection of 923 sputum samples from four countries (Ethiopia, Nepal, Nigeria, and Yemen) showed that the proportion of cases missed by the spot-morning and the spot-spot smears was not statistically different [2]. In that study, 210/216 (97%) of specimens with more than one positive smear were identified by both the standard and front-loaded schemes. In a different study in Nigeria, same-day diagnosis and the internationally recommended approaches were used on 224 patients with chronic cough, and identified 44 and 45 of the 78 patients with positive cultures, respectively [5], and the authors concluded that it could be possible to diagnose TB in a single day by examining two spot specimens. Similar studies have been done in the past and arguments made that reducing the number of sputum samples and visits will help to optimize smear microscopy [5], [9], [11] and reduce loss to follow up. In the current study 20/221 (9%) of the patients sampled failed to return for the morning sample next day, and six of these 20 suspects turned out to be smear positive by the first sample, further highlighting the importance of same-day diagnosis of TB. Among the HIV positive patients, frontloading identified 59/79 (74.7%) culture positive samples while the standard approach identified 55/79 (69.6%). In the HIV sero-negative category, on the other hand, front-loading identified 48/110 (43.6%) culture positive samples compared to 45/110 (40.9%) by the standard approach. These results are different from findings in Abuja where 106/194 (55%) patients were HIV positive and only 9–11% of their smears were positive compared with 30–32% for HIV negatives [5]. The reason for the difference may be due to the fact that our samples were picked from participants at a TB/HIV clinic where patients had been recruited following a smear positive/HIV positive criterion.

Findings from our study indicate that frontloading based on smear examination of two same-day sputum samples has a similar performance as the standard approach. This technique can reduce the number of visits required per patient and therefore suitable for use in our setting so as to reduce time to completion of smear microscopy on suspected individuals. However, its performance must be prospectively evaluated to determine the impact on case finding under program conditions.
